# Can the establishment of national sanitary cities better resist the impact of COVID-19?

**DOI:** 10.3389/fpubh.2023.1041355

**Published:** 2023-02-27

**Authors:** Gan Tianqi, Zhang Chunyan, Shen Renjun, Li Bo

**Affiliations:** ^1^School of Economics, Central China Minzu University, Wuhan, China; ^2^Hubei Moderately Prosperous Society in all Respects Construction Research Institute, Wuhan, China; ^3^School of Economics and Business Administration, Central China Normal University, Wuhan, China

**Keywords:** COVID-19, National Sanitary City, public health, public health management, urban policy

## Abstract

The global spread of COVID-19 has led to profound reflection on building a global public health security system. This paper uses the urban data collected during the COVID-19 epidemic in China in 2020 to evaluate the effect of the National Sanitary City (NSC) policy on the prevention and control of that epidemic at different stages. We found that the NSC policy was able to curb the occurrence and transmission of the epidemic the epidemic effectively after controlling a series of factors such as urban characteristics, population mobility and pathogen transmission. Compared with non-NSCs, the NSCs were better able to control the number of infected people and the infection rate and transmission rate, and this performance was even more impressive when the epidemic gradually entered the sporadic distribution stage. The heterogeneity analysis shows that the impact of the NSC policy on the prevention and control of COVID-19 differs according to the economic development level and population size. To a certain extent, the NSC policy has blocked the spread of viruses by continuously improving the urban medical and health system and strengthening the publicity concerning infectious disease prevention and control knowledge.

## 1. Introduction

At the end of 2019, the outbreak and spread of COVID-19 made an unprecedented impact on human health and global economic and social development (1). In sharp contrast to other countries in the world, China became the first major economy to achieve positive economic growth after the epidemic. The effective control of the epidemic requires not only the unified action of the whole country but also the modern public health system, with the foundation of infectious disease prevention, control and early warning. The construction of the NSC has eliminated the risk of Class A and B infectious diseases by improving the urban epidemic situation reporting network and improving the ability of medical institutions to prevent and control infectious diseases, which is an important policy affecting the construction of China's urban medical and health system. Therefore, we may ask: Did the NSCs make a difference during the outbreak of the COVID-19 epidemic?

In China, after the outbreak of COVID-19, the Chinese government issued a level-1 response to public health emergencies and took measures to initiate the immediate “lockdown of the city.” In the case of insufficient medical conditions in Wuhan, the spread of the epidemic was controlled in a short time by establishing “the hospital of Huoshen Mountain,” “the hospital of Leishen Mountain” and the “mobile cabin hospital,” to increase the quantity of medical beds (2), and by allocating medical staff to support Hubei and other areas. After that, China achieved “early detection, early isolation and early treatment” through vaccination, epidemic reporting and flow investigation.

In fact, as early as the 1950s, China established the Central Epidemic Prevention Committee (CEPC; later renamed the National Patriotic Health Campaign Committee) to manage and control epidemic prevention. After years of development, the NSCs managed and selected by the CEPC have become one of the most authoritative and wide-ranging city brand policies in China. The selection of NSCs follows the “NSC Standards” and “NSC Evaluation and Management Measures,” which have extremely strict requirements for urban medical and health development, especially in the field of infectious disease prevention and treatment. Since the city of Weihai in Shandong Province became the first NSC, the construction of the NSC has taken place over more than 30 years. By the end of 2021, 318 cities were included in the NSCs list. Therefore, it is of great significance to evaluate the actual effect of the NSC policy on the COVID-19 epidemic; moreover, it is very important to develop a modern medical system and explore China's epidemic prevention experience. At the macro level, this paper dialectically discusses the performance of NSC construction in the prevention and control of COVID-19, while on the micro level, it empirically analyzes the effect of the “sports” city brand construction.

The article structure arrangement is as follows: after the literature review in the second part, the third part introduces the prevention and control of COVID-19 in China in 2020, and the fourth part is an empirical analysis of the performance of the NSC policy in the fight against COVID-19, including a robustness test and heterogeneity analysis. Finally, we move on to the conclusion, policy enlightenment and research prospects.

## 2. Literature review

In order to deal effectively with the potential risks of the COVID-19 epidemic, mandatory short-term intervention measures became the main means adopted by governments before the vaccine became available. Isolation (3, 4), curfews (5), the closure of schools and public places (6, 7), traffic control (8, 9), paid sick leave (10) and a series of other policies were introduced to prevent the spread of the epidemic by restricting the flow of people.

The outbreak has had a strong exogenous impact on the economic order and triggered a series of chain reactions. The most serious epidemics in history have caused a serious labor force shortage in their respective economies and societies (11, 12). Even if the mortality rate was controlled effectively, it would still have had a huge impact on the labor market (13), and the unemployment rate would have risen (14–16). It is difficult for the unemployed population to achieve effective health protection (17), which in turn promotes the spread of the epidemic. The impact on the labor supply will cause the reduction of household consumption expenditure in the short term (18–20) and also be transmitted to the capital market (21–23) and real estate market (24), resulting in the decline of stock and house prices.

In the long run, the epidemic has led to the interruption of transportation networks between regions (25, 26) and the restriction of import and export activities (27–29). Social resources need to be reconfigured to cope with new market risks. The reconstruction of the global industrial chain and supply chain has led to a new crisis in the transformation of the domestic manufacturing industry (30). The imbalance between labor supply and demand caused by the epidemic, the downturn of the consumer market (31), the shutdown of production and the interruption of the supply chain have aggravated the uncertainty of economic growth. According to the *World Economic Outlook* report of the International Monetary Fund (IMF), the epidemic has caused a loss of US $12 trillion to the global economy, and humankind is experiencing the worst economic recession since the Great Depression (32).

The impact of an epidemic is evident. Efficient urban governance provides solutions to deal with potential risks. In the early stages of an outbreak, the stability of food supplies is key to keeping people safe, with regional collaboration in emergencies (33) and community-led food supplies (34) safeguarding food for people in affected areas. With the deepening of an epidemic, addressing the public's psychological panic and formulating precise prevention and control measures can effectively block the spread of that epidemic. Local governments mobilize community workers to participate extensively in epidemic prevention publicity, through mutual supervision between neighbors (35) and formulating propaganda slogans (36). Some extreme measures are even taken (37), to alleviate the public's psychological anxiety and ensure trust in the government (38). With the development and application of big data technology, some cities have promoted smart city projects (39) and developed special APP for epidemic prevention and control (40) to shorten the communication distance between people and the government. The application of these series of passive measures has alleviated the spread of the COVID-19 epidemic to a certain extent. However, for epidemic control, improving the medical and health level is the only way to improve the situation (41). In addition, city size and urban population density (42), governance capacity (43), urban planning (44) and other factors have influenced the spread of the COVID-19 epidemic.

In the existing literature, there are theoretical and empirical studies on the factors that have affected the spread of the COVID-19 epidemic and its impact on economic and social development. However, before the outbreak of the epidemic, how to effectively build an advanced urban medical and health system to deal with potential epidemics was already recognized as a problem deserving of consideration by global urban governance scholars. Therefore, we comprehensively evaluate the performance of China's “National Health Urban Governance” policy in response to the COVID-19 epidemic, with a view to providing a feasible plan for the construction of a modern health system.

## 3. NSC policy background and basic characteristics of COVID-19 in 2020

### 3.1. The NSC policy background

Since the founding of modern China, the country has experienced several regional epidemic diseases. In 1988, when the Shanghai epidemic broke out, 310,000 people in the city of 12.5 million were infected with hepatitis A in just 5 months. In the following year, the sixth meeting of the Standing Committee of the People's Republic of China deliberated and adopted a law on the prevention and control of infectious diseases. After experiencing SARS in 2003, H_7_N_9_ avian influenza in 2013, and COVID-19, the law of the People's Republic of China on the prevention and control of infectious diseases has been revised three times according to the findings of anti-epidemic work. The prevention and control mechanism of major infectious diseases should not only include the improvement of laws and regulations after the event, but also include the construction of the anti-epidemic defense line of the medical and health system. After years of development, China has established a screening technology system to identify more than 300 known and unknown pathogens within 72 hours of their discovery and a process for diagnosing, preventing and treating infectious diseases, contributing a number of “Chinese solutions” to the world.

The NSC is the highest honor of urban health management in the country. It is an acknowledgment of a city's tier classification and level of health. The selection of the NSC has carried out the stated titular aims of the Law of the People's Republic of China on the Prevention and Treatment of Infectious Diseases. It has assigned supervisors for infectious disease management according to regulations, carried out supervision and management according to law and carried out designated supervision of infectious disease supervision institutions and medical institutions of participating cities. Through the establishment of NSC Action, the NSC's infectious disease reporting network, prevention and health organizations, nosocomial infection control and epidemic registration and reporting systems have been comprehensively improved. The importance of these systems has been highlighted since the outbreak of COVID-19.

### 3.2. The characteristics of COVID-19 in 2020

**Figure 1:**
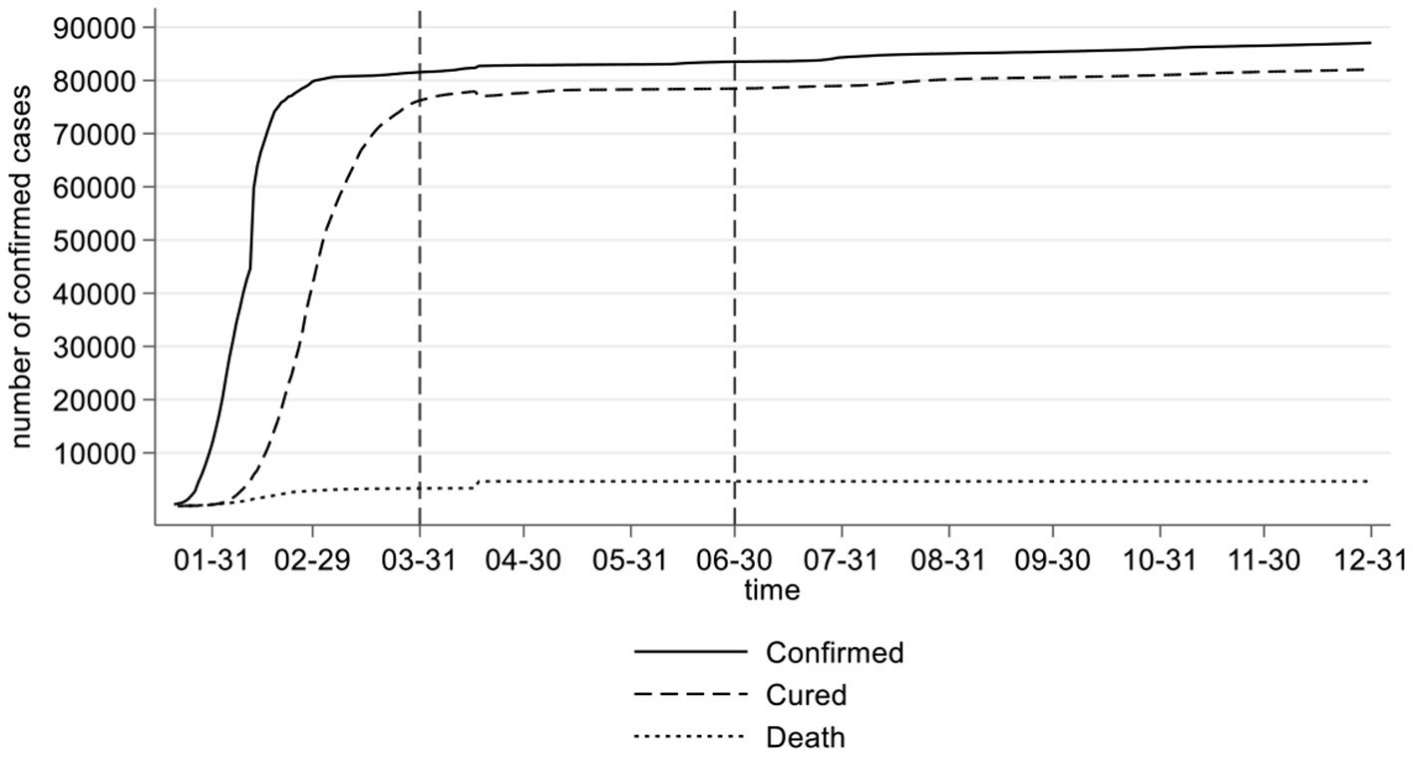
Chinese COVID-19 data in 2020.

Compared with the SARS epidemic in 2003, the COVID-19 epidemic has the characteristics of atypical onset and patients with a latent period. Asymptomatic infections can also become infectious sources. From the perspective of the epidemic situation, novel coronavirus is generally susceptible to people and has a long infection period. The infectious period includes an unknown incubation period, a conservatively estimated 20-day cure period and 14-day isolation observation period. If it is not closed and isolated during this period, the virus may spread through droplets, close contact and aerosols. As shown in Figure 1, during 2020, China experienced three stages in the fight against COVID-19. The first stage was the outbreak growth period (from January 23 to March 31, 2020). During this period, China implemented the containment policy, and the number of infected cases increased rapidly. As of March 31, 2020, a total of 81,554 cases had been confirmed nationwide. The second stage was the stable control period (from April 1 to June 30, 2020). During this period, the sealing and control policy was gradually implemented, and the new cases of infection were controlled. A total of 1980 cases were confirmed in this period. The third stage is the sporadic period (from July 1 to December 31, 2020), during which the local small-scale epidemic situation in the country was quickly and effectively controlled, and the number of deaths in this period achieved zero growth.

The key measures for epidemic prevention and control are to isolate pathogens and provide timely medical treatment. Adequate medical infrastructure and medical personnel have become the most important resources. Early diagnosis and isolation can cut off the transmission route of the virus and prevent further spread of the epidemic effectively. At the same time, under the treatment of medical personnel and professional equipment, it can be ensured that the patient is free from danger to their life, and the possibility of a cure is also greatly improved. The NSCs policy has formulated clear requirements in the field of infectious disease prevention and control. In the process of “creating NSCs”, the city is bound to strengthen medical and health construction and improve the corresponding ability of infectious disease prevention and control.

After the outbreak of COVID-19, the early treatment for the virus was still in the exploratory stage. The virus strain was successfully isolated on January 24, 2020. In February of the same year, animal experiments were begun. In June, they officially entered the clinical research experiment phase. In response to this global and large-scale outbreak, general medical facilities and a basic emergency warning system have not been able to provide complete support for epidemic prevention and control, which also leads to the continuous spread of COVID-19. The professional medical personnel, medical beds and equipment have had heterogeneous effects in controlling the occurrence and spread of the disease at various stages of the epidemic. Therefore, facing the outbreak of the epidemic, the rapid isolation of patients and emergency action of the medical system to prevent its spread are of equal importance. To investigate the prevention and control ability of a city to face an outbreak, we should not only observe the number of confirmed cases but also study the confirmed rate, transmission rate and death rate of COVID-19 in each period, especially based on the comprehensive consideration of the city's size and city's size and economic characteristics.

## 4. Performance of the NSCs in fighting against COVID-19

### 4.1. Model construction

In order to explore the performance of the NSCs in fighting against COVID-19, it is extremely necessary to identify whether there was a significant difference between NSCs and non-NSCs during the outbreak period. We selected cross-sectional data from 282 cities in China to explore the causal relationship between the anti-epidemic performance and “creating NSCs”. In the face of the explosive spread of COVID-19 in early 2020, the epidemic warning mechanism of each city was activated urgently, and the three epidemic prevention stages, respectively, in 2020 tested the capabilities of urban epidemic prevention. We captured epidemic data for each city at different times in 2020, including the number of people diagnosed, cured, and who died each day. The accumulated data are from March 31, June 30 and December 31, 2020, which are used as the main epidemic prevention and control indicators through data feature screening. Among the 282 cities included in the total sample, 160 cities were rated as NSCs before 2020, and the remaining 122 cities were analyzed as control samples. The model settings are as follows:


(1) $$Y_{i}=\beta_{0}+\beta_{1}treated_{i}+\beta_{2}\sum control_{i}+\theta_{i}$$

In Equation (1), *Y*_*i*_ is the explained variable; it mainly measures the various epidemic indexes of the city. We took the cumulative number of confirmed cases, transmission and mortality in the three stages of the “outbreak growth period”, “stable control period” and “sporadic period” in 2020 as the three indicator categories. *treated*_*i*_ is a dummy variable, indicating whether it is an NSC (yes, treated = 1; no, treated = 0), and if β_1_ is significant, it indicates that the NSCs had a positive impact on COVID-19 epidemic prevention. θ_*i*_ is random error term.

The advantage of using the cross-sectional data in this paper is that it allows us to unify the data before and after the outbreak of the epidemic and avoid structural errors in the process of statistical analysis. In order to study the net impact of the national health city policy in the model, observable information at the city level should be controlled to prevent the endogenous problems caused by the missing variable bias.

### 4.2. Control variables

As the influencing factors of epidemic prevention and control are relatively complex, we set four types of influencing factors as control variables in the regression analysis: urban scale, pathogen transmission, mobile transmission and urban characteristics, in order to strip out the real impact of the “NSC” on COVID-19 epidemic prevention and control. The control variables set (_*i*_) are as follows.

#### 4.2.1. Pathogen transmission

Although the source of the virus is still under exploration, Wuhan is the first city in China where COVID-19 was discovered and spread; thus, the distance between each city and Wuhan (*dist*) is used to measure the pathogen transmission. We need to control the space–time distance factor of virus transmission because Wuhan is the first disclosed city. Excluding the inflow from abroad, during the growth phase of the outbreak in the first quarter of 2020, the infections of the national epidemic were mostly carried by the outflow personnel in the city of Wuhan. Therefore, the space–time distance from Wuhan is the key factor for the spread of the virus.

#### 4.2.2. Mobile transmission

The existing research shows that cities with a high population density result in higher transmission speed (41). The mobile transmission is measured by the outflow of the population in each city and the inflow of the population within the city. We need to control the impact of the mobile transmission of virus carriers. Since January 24, after Wuhan announced the “lockdown of the city”, the number of confirmed cases increased significantly. Although the intensity of population flow decreased rapidly after the lockdown, the Spring Festival holiday led to a continuous increase in the intensity of population flow throughout the country, thus inevitably deepening the scope and speed of virus transmission.

#### 4.2.3. City characteristics

The city attribute is the key factor affecting the spread of the epidemic (43). The impact of the epidemic situation and overseas input is measured by whether the city is a frontier or an important seaport. The traffic attribute is measured by the air passenger volume level (*airport*), and the influence of administrative differences on urban epidemic prevention is controlled by whether it is a provincial capital city (*prov_capi*).

#### 4.2.4. City size

The cities with high population density have greater difficulties in controlling the spread of the epidemic (41). The city size is measured by the urban population density (*pop*), which mainly controls the number of confirmed cases and virus infections caused by population factors. To a certain extent, the scale of the city reflects the characteristics of the city's economy, transportation, population and residence, and also has an important influence on the possibility of outbreak and transmission.

### 4.3. Data

We collected data from 282 cities; they include three stages and three types of epidemic prevention indicators as explained variables, two indicators (an NSC or the number of years it has been an NSC) as the explanatory variables and eight control variables that may affect the spread of the epidemic. The calculation formula of the explained variables is shown in Table 1, which mainly includes 12 indexes: the number of confirmed cases, the rate of confirmed cases, the transmission rate and the mortality rate. All indicators were normalized in the empirical process to eliminate the impact of variable dimensions. The specific formula is Formula (2). Since the number of confirmed cases in the base period cannot be zero, in the calculation formula of epidemic transmission indicators, the denominator was thus set as the number of confirmed cases at the end of January.


(2) $$\begin{array}{l} x^{{}^{\prime }}=\frac{\left(x-x_{\min}\right)}{\left(x_{\max}-x_{\min}\right)} \end{array}$$

**Table 1:** Descriptive statistics.

	**Variables**	**Measurement method**	**Mean**	**Min**	**Max**
Explained variable	*conf*3	Cumulative number of confirmed cases (up to March 31)	286.858	1	50,007
	*conf*6	Cumulative number of confirmed cases (up to June 30)	293.213	1	50,340
	*conf*12	Cumulative number of confirmed cases (up to December 30)	304.408	1	50,354
	*conf*_*R*3	$\frac{conf3}{TotalPopulation}$	0.488	0.004	55.195
	*conf*_*R*6	$\frac{conf6}{Total\;Population}$	0.499	0.004	55.563
	*conf*_*R*12	$\frac{conf12}{Total\;Population}$	0.522	0.004	55.578
	*spre*3	$\frac{confirmed\;cases\;\left(from\;31\;Jan\;to\;31\;Mar\right)}{confirmed\;cases\;\left(up\;to\;31\;Jan\right)}$	2.619	0.000	25.000
	*spre*6	$\frac{confirmed\;cases\;\left(from\;31\;Jan\;to\;30\;Jun\right)}{confirmed\;cases\;\left(up\;to\;30\;Jun\right)}$	2.997	0.000	66.833
	*spre*12	$\frac{confirmed\;cases\;\left(from\;31\;Jan\;to\;31\;Dec\right)}{confirmed\;cases\;\left(up\;to\;31\;Dec\right)}$	3.565	0.000	104.625
	*death*_*R*3	$\frac{Death\;of\;cases\;\left(up\;to\;31\;Mar\right)}{confirmed\;cases\;\left(up\;to\;31\;Jan\right)}$	0.009	0.000	0.250
	*death*_*R*6	$\frac{Death\;of\;cases\;\left(up\;to\;30\;Jun\right)}{confirmed\;cases\;\left(up\;to\;30\;Jun\right)}$	0.009	0.000	0.250
	*death*_*R*12	$\frac{Death\;of\;cases\;\left(up\;to\;31\;Dec\right)}{confirmed\;cases\;\left(up\;to\;31\;Dec\right)}$	0.009	0.000	0.250
Explanatory variable	*treated*	NSC, treated=1; non-NSC, treated=0	0.564	0	1
	*age*	Years of being an NSC	6.099	0	30
Control variable	*pop*	Population density	0.046	0.001	0.276
	*Lndist*	Distance from Wuhan	13.631	0	15.044
	*flow*_*out*	Intensity of population migration in and out	3.665	0.249	28.299
	*flow*_*in*	Population flow intensity in the city	5.455	2.437	7.678
	*frontier*	1, Open border cities	0.007	0	1
	*seaport*	1, Important seaport cities	0.085	0	1
	*airport*	Air passenger capacity level	1.610	0	5
	*prov*_*capi*	1, Provincial capital	0.092	0	1

Among the control variables, the urban population density (*pop*) was calculated by the ratio of the population at the end of 2019 to the urban administrative area. The space–time distance (*lndist*) from Wuhan was obtained by crawling the data of the shortest driving time between the cities in the Gaode map. The population flow intensity (*flow out*) comes from the Baidu Migration Index Database (BMID). We calculated the average daily immigration and emigration intensity of each city in the week before the “lockdown of the city”. The air passenger volume grade (*airport*) was determined by the air passenger volume data of each city in 2019. We divided the urban passenger volume into six grades according to the quantile.

### 4.4. Empirical results

According to the differential performance of cities in resisting epidemic outbreaks, we constructed epidemic prevention indicator data sets in different periods to explore whether the NSCs had better performance during the COVID-19 epidemic. The regression results are shown in Table 2. Overall, under the control of other unchanged factors, the three periods with the number of confirmed cases and the rate of confirmed cases as the dependent variables, the treated were significantly negative. The transmission coefficient in the three periods is negative, but only significant in the sporadic stage, while the coefficient of mortality was not significant.

**Table 2:** Regression results.

	**(1) conf3**	**(2) conf6**	**(3) conf12**	**(4) conf_R3**	**(5) conf_R6**	**(6) conf_R12**
*treated*	−0.010^**^ (−2.13)	−0.010^**^ (−2.14)	−0.011^**^ (−2.17)	−0.446^*^ (−1.88)	−0.469^*^ (−1.95)	−0.502^**^ (−2.05)
*lndist*	−0.049^***^ (−3.33)	−0.049^***^ (−3.39)	−0.049^***^ (−3.38)	−2.975^***^ (−4.63)	−2.988^***^ (−4.60)	−2.973^***^ (−4.57)
*flow*_ out*in	−0.001^**^ (−2.28)	−0.001^**^ (−2.29)	−0.001^**^ (−2.30)	−0.093^***^ (−2.83)	−0.095^***^ (−2.86)	−0.097^***^ (−2.88)
*flow*_*out*	0.002^*^ (1.84)	0.004^*^ (1.87)	0.004^*^ (1.91)	0.240^**^ (2.37)	0.246^**^ (2.11)	0.251^**^ (2.13)
*flow*_*in*	−0.007^*^ (−1.79)	0.007^*^ (−1.79)	0.007^*^ (−1.79)	−0.465^**^ (−2.31)	−0.468^**^ (−2.30)	−0.486^**^ (−2.36)
*frontier*	0.045^***^ (3.12)	0.045^***^ (3.12)	0.045^***^ (3.12)	2.410^***^ (3.65)	2.420^***^ (3.63)	2.416^***^ (3.62)
*seaport*	0.005 (1.15)	0.005 (1.13)	0.005 (1.20)	0.275 (1.12)	0.253 (1.00)	0.273 (1.06)
*airport*	0.004^**^ (2.26)	0.004^**^ (2.29)	0.004^**^ (2.30)	0.161^**^ (2.03)	0.176^**^ (2.12)	0.184^**^ (2.21)
*prov*_*capi*	0.020^**^ (2.16)	0.020^**^ (2.16)	0.021^**^ (2.25)	1.164^**^ (2.47)	1.147^**^ (2.39)	1.309^**^ (2.51)
	**(7) spre3**	**(8) spre6**	**(9) spre12**	**(10) death**_**R3**	**(11) death**_**R6**	**(12) death**_**R12**
*treated*	−0.425 (−1.18)	−1.346 (−1.65)	−2.464^**^ (−2.02)	0.002 (0.52)	0.002 (0.46)	0.002 (0.44)
*pop*	1.507 (0.22)	−0.215 (−0.03)	−2.865 (−0.26)	−0.030 (−0.50)	−0.034 (−0.57)	−0.032 (−0.53)
*lndist*	−0.567^**^ (−2.48)	−0.426 (−1.43)	0.036 (0.07)	−0.002 (−0.94)	−0.003 (−1.47)	−0.003 (−1.52)
*flow*_ out*in	−0.050^*^ (−1.94)	−0.078^*^ (−1.79)	−0.101 (−1.41)	−0.000 (−0.24)	0.000 (−0.38)	−0.000 (−0.50)
*flow*_*out*	0.150 (1.55)	0.214 (1.47)	0.139 (0.64)	−0.000 (−0.06)	0.000 (0.02)	0.000 (0.08)
*flow*_*in*	0.155 (0.74)	−0.019 (0−0.06)	−0.678 (−0.90)	−0.002 (−0.056)	−0.003 (−0.58)	−0.002 (−0.55)
*frontier*	−0.953^***^ (−2.67)	−1.123^**^ (−2.48)	−0.850 (−1.31)	−0.012^**^ (−2.48)	−0.010^**^ (−2.21)	−0.010^**^ (−2.22)
*seaport*	−0.225 (−0.33)	−1.312 (−1.08)	−0.532 (−0.33)	0.004 (0.56)	0.004 (0.61)	0.004 (0.60)
*airport*	−0.047 (−0.38)	0.530 (0.98)	−0.860 (1.52)	−0.002^**^ (−2.23)	−0.002^**^ (−2.14)	−0.002^**^ (−2.13)
*prov*_*capi*	0.959 (1.42)	0.011 (0.01)	4.409 (0.95)	0.007^*^ (2.14)	0.007^*^ (2.15)	0.007^*^ (2.12)
Obs	282	282	282	282	282	282

T-value in parentheses,

*** p < 0.01,

** p < 0.05,

* p < 0.1.

The results showed that compared with non-NSCs, NSCs showed better effects in suppressing the number of confirmed cases and the rate of confirmed cases. There are two potential reasons for the positive but not significant mortality rate. First, in the period of explosive growth, the sharp increase of infected patients overloaded the medical system, making it unable to take mild patients into account effectively. In addition, neither a specific drug nor an effective treatment and prevention means for COVID-19 had been found anywhere globally. Second, according to the death cases data, most of the patients who died of COVID-19 had basic diseases, and COVID-19 may be one but not the only cause of death. Therefore, the mortality rate will not be further discussed in the following text.

The results show that when the number of confirmed cases is taken as the explained variable, the performance of the treated coefficient is relatively stable. When the diagnostic rate is taken as the explained variable, the treated coefficient gradually increases. When the transmission rate is taken as the explained variable, the treated coefficient is significantly negative only in the sporadic stage. This series of evidence shows that compared with non-NSCs, NSCs could better transform the foundation by becoming advantageous against COVID-19 before it could be effectively brought under control. After the epidemic entered the sporadic stage, there were more prominent performances in controlling the number of infections and infection rate, as well as in curbing the spread of the epidemic.

The results of the control variables show that the closer to Wuhan and the border area, the greater the air-carrying capacity; furthermore, the provincial capital cities bear more epidemic prevention pressure, especially in the sporadic stage, and consequently these cities have a higher incidence of epidemic. The coefficient of population mobility shows that the coefficient of mobility intensity between cities is significantly positive and increasing. It indicates that the increased mobility between cities will increase the incidence of the epidemic. This positive effect is the strongest in sporadic stage.

### 4.5. Robustness test

#### 4.5.1. Eliminate interference from National Civilized City (NCC) policy

Civilization and health are the theme of the sustainable development of urban construction. Since the twenty-first century, various urban brands have emerged on the track of building advanced cities in China. Many cities have set the strategic goal of creating a “National Civilized City”(NCC) and “National Sanitary City”(NSC), and some cities have even sounded the construction slogan of “co creation of five city brands” and “co creation of six city brands”. According to the data, we found that the NSCs partly overlapped with the NCCs in the total sample. Therefore, in order to verify the reliability of the results, we must exclude the interference of the NCC policy.

First, we removed the NSC samples from the total sample, so that the remaining samples were composed of NCCs and other cities without these two kinds of attributes. Then, we set NCC = 1 for the NCCs sample and 0 for the rest to replace treated into the model (1) for empirical analysis. If the NCC coefficient was not significant, it indicated that the previous empirical results were not affected by the NCC policy. As shown in Table 3, the regression results of various indicators are no longer significant after taking the NCC policy as independent variables. It proves that the NCC policy has nothing to do with the epidemic prevention effect in the various periods of COVID-19.

**Table 3:** Influence of NCC policy.

	**(1) conf3**	**(2) conf6**	**(3) conf12**	**(4) conf_R3**	**(5) conf_R6**
*NCC*	−0.001 (−0.85)	−0.001 (−0.90)	−0.0002 (−0.13)	−0.153 (−1.08)	−0.183 (−1.24)
*countrol*	YES	YES	YES	YES	YES
*Obs*	116	116	116	116	116
	**(6) conf**_**R12**	**(7) spread3**	**(8) spread6**	**(9) spread12**	
*NCC*	0.022 (0.09)	0.223 (0.26)	−1.633 (−0.92)	3.812 (0.70)	
*countrol*	YES	YES	YES	YES	
*Obs*	116	116	116	116	

T-value in parentheses, ^***^p < 0.01, ^**^p < 0.05, ^*^p < 0.1.

#### 4.5.2. Quantile regression test

**Figure 2:**
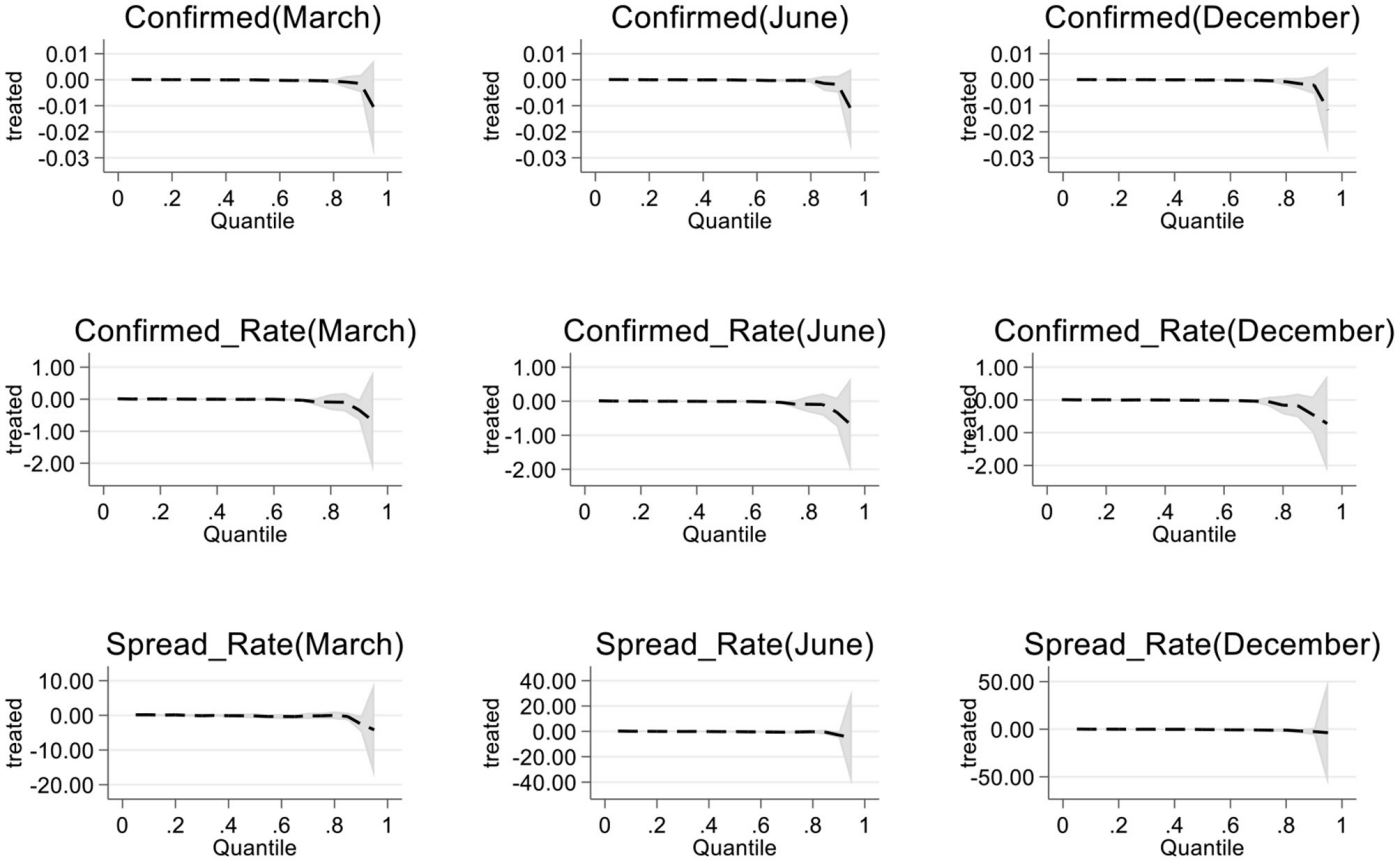
Quantile regression results.

Due to the different space–time distances, the occurrence and transmission of epidemic diseases in cities are quite different. Therefore, we used quantile regression to test the results of the model. Figure 2 shows the regression results in terms of the cumulative confirmed number, cumulative confirmed rate and cumulative transmission rate at different stages. The results based on the cumulative number of confirmed cases were significant at all loci, and the influence effect of samples from 0 to 80 loci was not significant. In the three stages, the effect of the cumulative diagnosis rate at <90th percentile was not obvious. There is a significant effect at loci with a cumulative transmission rate of <80, and the results are relatively robust. This shows that there is a “fault effect” between cities with a serious epidemic and other cities, and national health cities with an extremely serious epidemic do not have outstanding epidemic prevention and control capabilities; however, this does not affect the main conclusions of the full text.

### 4.6. Heterogeneity analysis

#### 4.6.1. Divide the city sample

**Figure 3:**
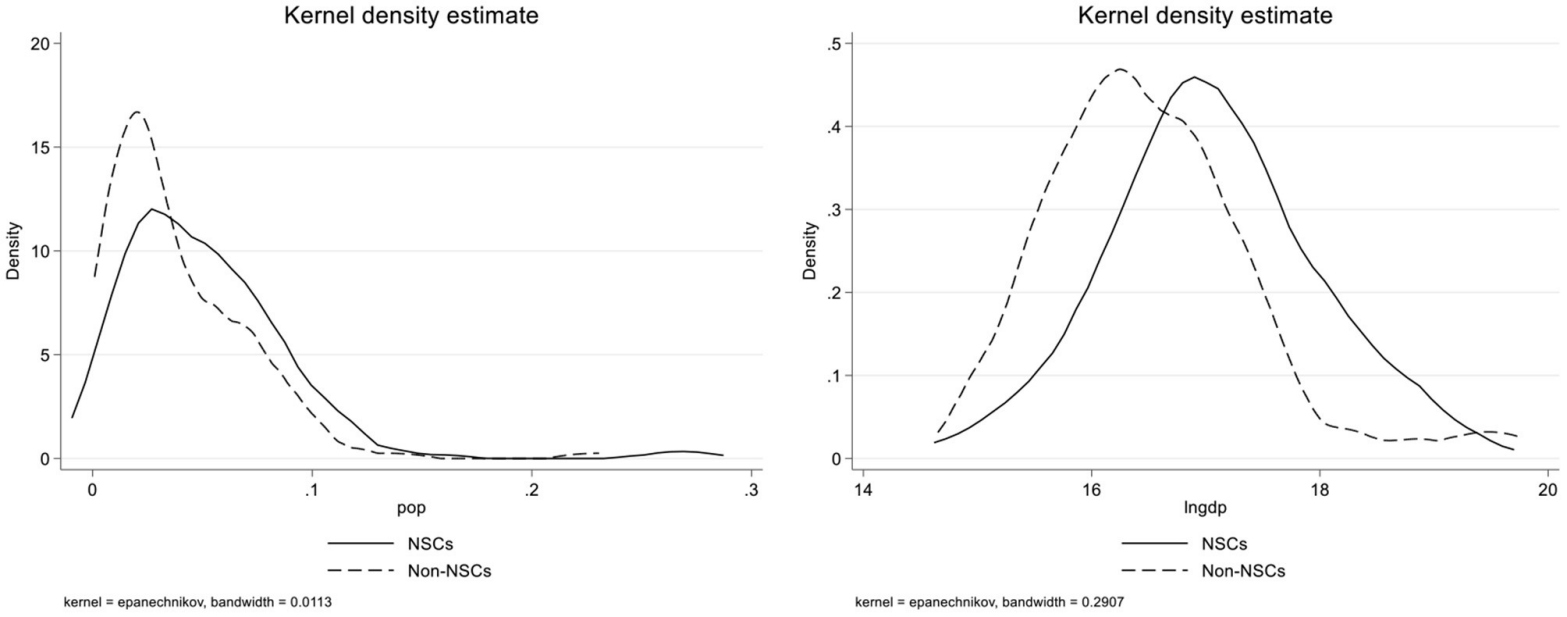
Distribution of urban population density and economic level nuclear density.

We have proved that NSCs demonstrate outstanding performance in curbing the occurrence and spread of the COVID-19 epidemic, but the epidemic prevention capacity of NSCs is different in various stages and indicators. As shown in Figure 3, by drawing the nuclear density map of NSCs and non-NSCs, we found that the economic level and city size of NSCs are different from other cities. The population density of non-NSCs is more concentrated, while the economic development level of NSCs is better. To explore the heterogeneity of the results, we divided the samples according to economic level and population size and then judged whether or not there were differences in the impact of “creating NSC” on epidemic resistance under different urban characteristics.

We considered four types of epidemic indicators, but only the performance of transmission rate in each stage is different. Therefore, in the heterogeneity analysis, we mainly discussed the epidemic transmission in three stages. As shown in Table 4, a better economic level and higher population density of a large city mean that the NSC's influence on the three stages of epidemic prevention and control effect is not significant. In the samples with poorer economies and lower population density, the impact effects of NSCs in the three stages are different. Among them, the NSCs with a poorer economy inhibited the further spread of the epidemic in the second and third stages significantly, and the impact effect of the third stage was greater than that of the second stage. The NSCs with lower population density have a significant inhibitory effect only at the sporadic stage of the epidemic. To sum up, the influence of the NSC policy on epidemic prevention and control is inconsistent under different economic levels and city sizes. In the normalization stage, the NSCs with poorer economies and smaller city sizes show better epidemic prevention and control capabilities.

**Table 4:** Heterogeneity analysis of different types of NSCs.

	**Better economy**			**Poor economy**		
	**(1) spread3**	**(2) spread6**	**(3) spread12**	**(4) spread3**	**(5) spread6**	**(6) spread12**
*treated*	0.601 (0.73)	−0.598 (−0.39)	−8.399 (−1.17)	−0.556 (−1.51)	−1.156^*^ (−1.66)	−1.388^*^ (−1.92)
*countrol*	YES	YES	YES	YES	YES	YES
*obs*	87	87	87	195	195	195
	**Large population density**			**Low population density**		
	**(7) spread3**	**(8) spread6**	**(9) spread12**	**(10) spread3**	**(11) spread6**	**(12) spread12**
*treated*	0.066 (0.11)	−0.452 (−0.55)	−0.476 (−0.48)	−0.642 (−1.55)	−1.724 (−1.58)	−3.417^*^ (−1.90)
*countrol*	YES	YES	YES	YES	YES	YES
*obs*	114	114	114	168	168	168

T-value in parentheses, ^***^p < 0.01, ^**^p < 0.05,

* *p* < 0.1.

#### 4.6.2. Replace explanatory variable

To explore whether there were differences in the years of becoming an NSC, we replaced the dummy variable of whether it is an NSC with the number of years it has been an NSC. The results are shown in Table 5: the coefficients in columns (1)–(6) are significantly negative, and the years of being an NSC have a significant impact on the number of confirmed cases and the rate of confirmed cases. The coefficients in columns (7)–(12) are negative but not significant, so the transmission rate and mortality rate of the epidemic are not affected. This indicates that the earlier the establishment of an NSC, the better it performs in curbing the occurrence of the epidemic, but it does not affect the spread of the epidemic. We found that the NSC policy has a certain cumulative effect in suppressing the outbreak of the epidemic.

**Table 5:** Regression results of changing independent variables.

	**(1) conf3**	**(2) conf6**	**(3) conf12**	**(4) conf_R3**	**(5) conf_R6**	**(6) conf_R12**
*age*	−0.014^**^ (−1.98)	−0.014^**^ (−2.01)	−0.014^**^ (−2.03)	−0.790^**^ (−2.10)	−0.831^**^ (−2.17)	−0.877^***^ (−2.24)
*countrol*	YSE	YSE	YSE	YSE	YSE	YSE
*Obs*	282	282	282	282	282	282
	**(7) spread3**	**(8) spread6**	**(9) spread12**	**(10) death**_**R3**	**(11) death**_**R6**	**(12) death**_**R12**
*age*	−0.556 (−0.80)	−2.203 (−1.46)	−3.452 (−1.53)	−0.000 (−0.05)	−0.001 (−0.10)	−0.001 (−0.19)
*countrol*	YSE	YSE	YSE	YSE	YSE	YSE
*Obs*	282	282	282	282	282	282

T-value in parentheses,

*** *p* < 0.01,

** *p* < 0.05,

^*^p < 0.1.

## 5. Conclusions and policy implications

### 5.1. Conclusions

The COVID-19 epidemic has swept the world and made a tremendous impact on social development and human survival, both of which cannot be separated from the protection of social systems. In the face of the sudden impact of the epidemic, these systems became the best guarantee of survival. China has formulated an emergency early-warning mechanism to deal with outbreaks of the epidemic, plus follow-up normalized protection, and these cannot be separated from the training of the medical and health system and the efficient action of various departments at all levels. It has been 30 years since the NSC policy was issued, and the number of NSCs has reached 318. This policy has had a strong impact on the development of urban medical and health systems, especially in the field of infectious disease prevention and control.

We focused on the differences between cities with COVID-19 epidemic prevention and control and discussed the outstanding performance of NSCs at various stages, explored the effect of the NSC policy in suppressing the occurrence of the epidemic and drew the following conclusions:

1) NSCs demonstrated outstanding performance in the prevention and control of COVID-19 in 2020, and their performances in the outbreak growth period in the first quarter, the stable control period in the second quarter and the sporadic divergence period in the last two quarters are different. A series of robustness tests have proved the reliability of the results. The inhibitory effect against the occurrence of the epidemic is 1, 1, and 1.1% in the three stages, and the inhibitory effect against the incidence of the epidemic is 44.6, 46.9, and 50.2% in the three stages.
2) In the three stages, NSCs demonstrate significant differences in curbing the spread of COVID-19, and the effect is most obvious in the sporadic and divergent stages. The NSCs do not show an outstanding performance in curbing COVID-19 deaths, and there is no difference in the research of new virus treatments by medical systems in the short term.
3) The heterogeneity analysis shows that the NSCs with poorer economies or lower population density are more prominent in suppressing the spread of the epidemic. Moreover, the inhibition effect in cities with lower economic performance is highest in the sporadic stage, while cities with lower population density show a prominent inhibition effect in the sporadic stage.
4) There is a certain cumulative effect of experience in NSCs in curbing the occurrence of the epidemic, which reflects the effectiveness and incentives of the follow-up review mechanism of the NSC policy. This effect is one of the reasons why NSCs demonstrate better performance in COVID-19 epidemic prevention.

### 5.2. Policy implications

China has provided a high-quality sample of a process of COVID-19 prevention and control, providing a reference point for the global response to health emergencies. In the fight against the virus, the entirety of humankind should adhere to the principle of “life first”, in order to jointly build a community with a shared future that safeguards the Earth. The NSC policy, with continuous improvement of medical and health standards, has played a prominent role in the prevention and control of major infectious diseases, which reflects the progressiveness of China's strategic deployment in responding to sudden crisis events. The brand policy of the NSC, which is competitive, incentivizing and long-term, has injected great power and vitality into urban construction and development. At present, the prevention and control of COVID-19 has become normal, but the great impact of the crisis has sounded the alarm for the world. We should take more and more targeted actions to prevent unknown public health events.

1) We should continue to promote the construction of NSCs and continue to give play to the driving force of the NSC policy to improve urban medical and health levels. Most of the existing policies are oriented to the prevention and control measures introduced after the outbreak of the epidemic, and the cost of the *ex ante* policy is far less than the *ex post*. Building an effective early warning and response mechanism should be the guide of current urban governance.
2) We should improve the review standards in the field of infectious disease prevention and control when selecting NSCs. It is absolutely necessary to strictly follow the review standards and implement the withdrawal mechanism in the review process. Strict standards ensure the authority of the identification of the NSC, and the “exit mechanism” can play a good role in the long-term effect of the policy.
3) We can set up new tracks in urban construction and development strategies specifically for infectious disease prevention and control. The NSC policy includes the selection criteria for urban health, infectious disease prevention, pollution discharge and other fields. The establishment of a new city brand for infectious disease prevention can better improve the level of urban health care.

### 5.3. Prospects

This paper has comprehensively assessed the impact of the NSC policy on epidemic prevention and control in China, and combined with existing research, there is still room for expansion.

1) The dynamic data model and big data simulation can provide a broader perspective for evaluating the effectiveness of policies, which may also be a direction for evaluating the impact of COVID-19 in the future. At the same time, in view of the availability of data, the traditional compartment model in the field of infectious diseases cannot be used in this paper; thus, the policy evaluation of non-experimental data lacks reference in the medical field.
2) The epidemic situation is still developing. The policies of various countries and regions to deal with the epidemic situation are different. The use of dummy variables to reflect policy variables lacks consideration of the intensity of epidemic policy.

## Data availability statement

Publicly available datasets were analyzed in this study. This data can be found here: Please request by mail gantq826@126.com.

## Author contributions

All authors listed have made a substantial, direct, and intellectual contribution to the work and approved it for publication.
